# Alteration of Forest Structure Modifies the Distribution of Scale Insect, *Stigmacoccus garmilleri*, in Mexican Tropical Montane Cloud Forests

**DOI:** 10.1673/031.011.12401

**Published:** 2011-09-19

**Authors:** Heather A. Gamper, Suzanne Koptur, Jose García-Franco, Andres Plata Stapper

**Affiliations:** ^1^ Department of Biological Sciences, Florida International University, Miami, Florida 33 199, USA; ^2^ Department of Geography, Florida State University, Tallahassee, FL 32306, USA; ^3^ Departamento de Ecología Funcional, lnstituto de Ecología, Xalapa, Veracruz 91070, Mexico; ^4^ Department of Biological Science, Florida State University, Tallahassee, FL 32306, USA

**Keywords:** habitat fragmentation, land use change, Margarodidae, plant-animal interactions, Stigmacoccidae

## Abstract

*Stigmacoccus garmilleri* Foldi (Hemiptera: Margarodidae) is an ecologically important honeydew-producing scale insect associated with oak trees (*Quercus* spp.) in highland forests of Veracruz, Mexico. The honeydew exudates of *S. garmilleri* serve as a significant nutrient source to many species of birds, insects, and sooty molds. Oak trees found in the forest interior, forest edge, and those scattered in pasture areas support scale insect colonies, though the pattern of insect infestations on trees within these varying landscape types has not been elucidated. This study aims to describe the distribution of scale insect infestation and any distinctions in honeydew production based on tree location. Scale insect density, honeydew volume, and sugar concentration were surveyed throughout a continuous landscape that included both patches of forest and scattered pasture trees. In addition, the anal filament through which the honeydew drop is secreted was also measured and was experimentally removed to test and measure regrowth. Scale insect densities on tree trunks were greatest on pasture trees, while intermediate densities were found on trees at the forest edge, and low densities on interior forest trees, suggesting that trees in disturbed areas are more susceptible to scale insect infestation. Trees with small diameters at breast height had significantly higher insect densities than trees with medium to large diameters. Trunk aspect (North, South, East, and West) was not a significant determinant of scale insect density. In forested areas higher densities of scale insects were found at three meters height in comparison to lower heights. Sugar concentrations and drop volumes of honeydew in forest and pasture areas were not significantly different. However, scale-insect anal tubes/filaments were significantly longer in pasture than they were in forests. Sugar concentrations of honeydew appeared to be positively correlated with temperature and negatively correlated with relative humidity. Experiments indicated that anal filaments could grow approximately 4 mm every 24 hours, and average tube growth was significantly faster in pasture than in forest, suggesting that there may be a physiological effect on the insect due to landscape disturbance. The results obtained in this study describe the increases in scale insect infestation of trees with forest disturbance. The effect of these increased scale insect densities on the host tree physiology is still to be resolved.

## Introduction

The montane cloud forests of northeastern Mexico have a high concentration of endemism and are increasingly vulnerable to climate change, deforestation, and habitat fragmentation (Markham 1998; Bubb et al. 2004). Ninety percent of the original montane cloud forest in Mexico has been lost (Ramirez-Marcial et al. 2001). Remaining forests are discontinuously distributed and commonly form an abrupt boundary with adjacent grazed grassland, small agricultural fields, and clearings for houses and community structures. As habitat fragmentation becomes more pervasive throughout the world, our understanding of forest fragmentation has also grown more sensitive to context. The species interactions initiated with fragmentation and post-fragmentation disturbance regimes may magnify the impacts of fragmentation (Laurance 2002; Ewers and Didham 2006; Malanson et al. 2007).

**Figure 1.**
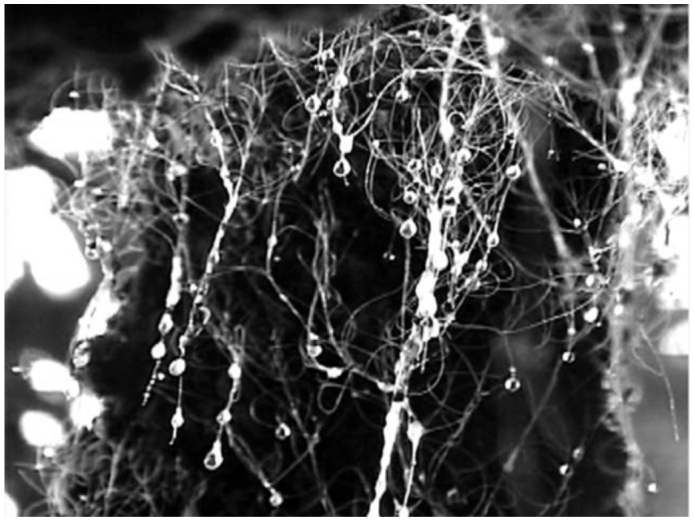
Hair-like anal filaments from scale insect *Stigmacoccus garmilleri* and drops of honeydew secreted from their ends (Chiconquiaco, Veracruz, Mexico). Scale insects are capable of reaching high densities on oak tree trunks and branches. High quality figures are available online.

**Figure 2.**
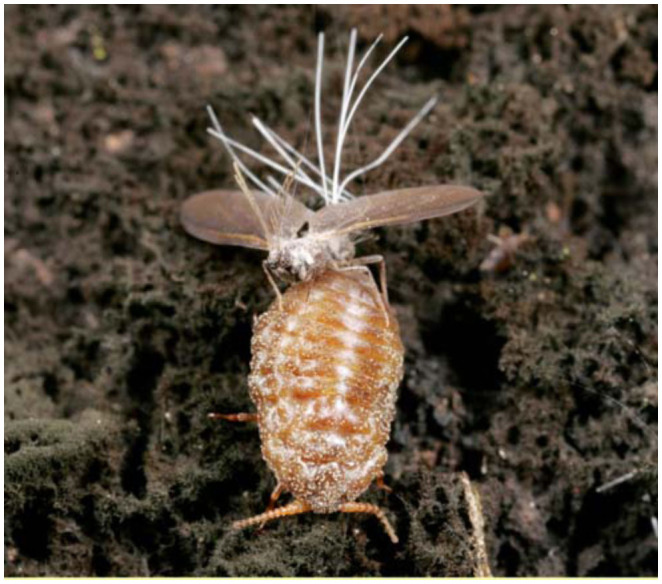
Winged adult male (*Stigmacoccus garmilleri*) mating with soft-bodied adult female. For approximately three weeks during their life cycle adults are found mating on the trunks of host trees (*Quercus* spp.) in tropical montane forests of central Veracruz, Mexico. High quality figures are available online.

A notable feature of the highly fragmented montane forests of central Veracruz, Mexico, is the interaction between oak trees (*Quercus* spp.) and phloem-feeding scale insects (Gamper and Koptur 2010), identified as *Stigmacoccus garmilleri* Foldi (Hemiptera: Margarodidae) (Foldi 1995; Hodgson et al. 2007). Immature *S. garmilleri* instars colonize trunks and branches by burrowing under tree bark. The scale insects insert their mouthparts, called stylets, into phloem cells and feed on phloem. The phloem of the host plant is rich in carbohydrates but low in compounds containing soluble nitrogen and amino acids, which are necessary to the insects for protein building (Gullan and Kosztarab 1997). Phloem feeding insects therefore ingest and excrete large quantities of carbohydrates in the process of acquiring sufficient amino acids (Wäckers 2000). This waste excretion, termed honeydew, forms droplets at the end of long anal tubes, or anal filaments (Figure 1). Honeydew-producing insects tend to eliminate copious honeydew, live in groups, and are typically sedentary or semi-sedentary (Williams and Williams 1980). For several months of the year *S. garmilleri* resides within the tree in the form of these feeding instars. Adult female and male insects develop and can be found mating on the surface of the tree (Figure 2). Details of the life cycle of *S. garmilleri* were described by Hodgson et al (2007).

**Figure 3.**
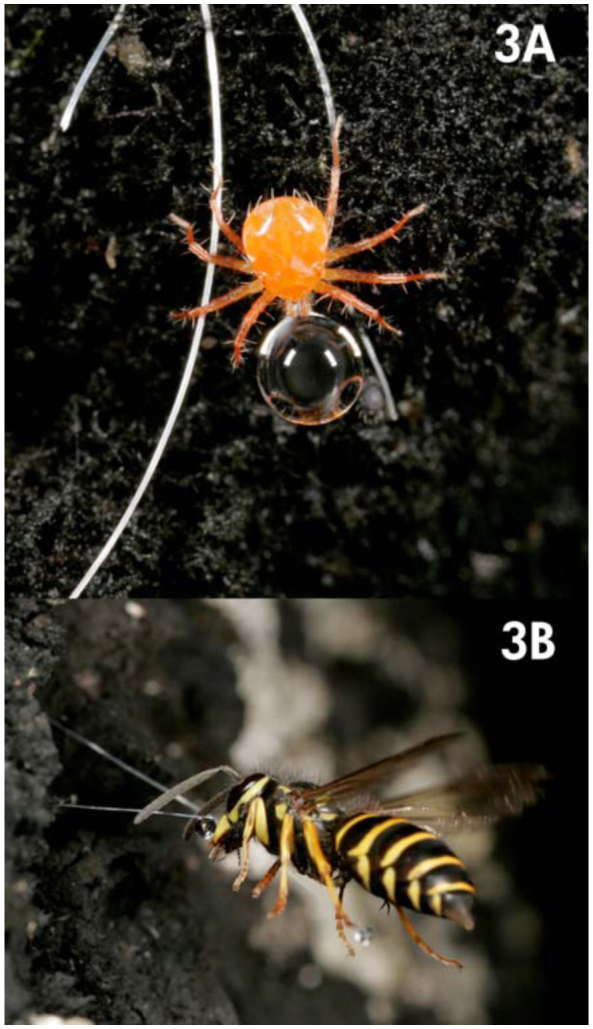
Invertebrate species are known to feed on honeydew produced by scale insect *Stigmacoccus garmilleri* in tropical montane forests of central Veracruz, Mexico, (a) Mite (*Anystis* sp.) found foraging on a honeydew droplet and (b) Vespid wasp in flight consuming honeydew. High quality figures are available online.

In some ecosystems, honeydew forms important ecological links within trophic levels and occasionally represents the primary carbohydrate food source for diverse group of organisms (Grant and Beggs 1989). Birds are commonly found foraging on and defending the rich and abundant honeydew resource originating from *S. garmilleri* (Gamper and Koptur 2010). The honeydew of *S. garmilleri* also provides food for a varied community of arthropods such as ants, vespid wasps (Figure 3A), honeybees (*Apis mellifera*), mites (*Anystis* sp.) (Figure 3B), and dipteran species. Furthermore, honeydew provides nourishment for a dense growth of black sooty mold that in turn provides habitat for many invertebrates (Didham 1993; HA Gamper unpublished data) and may provide nourishment for bacteria and fungi that decompose the litter on the forest floor (Morales 1991), although Wardhaugh and Didham (2006) found lower decomposition rates in high honeydew areas.

Despite the importance of honeydew in montane forests of Mexico, little is known about the distribution of scale insect colonies in the present landscape. Insects that are concealed while feeding, such as sap-sucking scale insects, stem-borers, and leaf miners, are likely to have heterogeneous spatial distributions in the host environment (Wardhaugh et al. 2006). Changes to the forest structure may alter the density and distribution of scale insects and the honeydew resource. Local factors such as tree species, tree size, growth rate, genotype, and exposure are believed to generate the large population variances observed between adjacent trees (Gaze and Clout 1983; Kelly 1990; Didham 1993). Individual trees can also be vertically stratified into discrete zones or layers from the roots to the crown (Didham and Fagan 2004). In New Zealand beech forests the within-tree distribution of the sooty beech scale was vertically stratified with the greatest insect densities occurring on bark surfaces in the canopy instead of on lower trunk surfaces (Wardhaugh et al. 2006).

The study presented here investigates populations of *S. garmilleri*, a scale insect whose stable persistence benefits an array of species in a Mexican tropical montane cloud forest habitat that has undergone considerable land-use change. The main objective of this work is to compare the distribution of scale insects and their honeydew production on the host tree in three distinct habitats, namely forest interior, forest edge, and isolated pasture.

## Materials and Methods

### Study site

**Figure 4.**
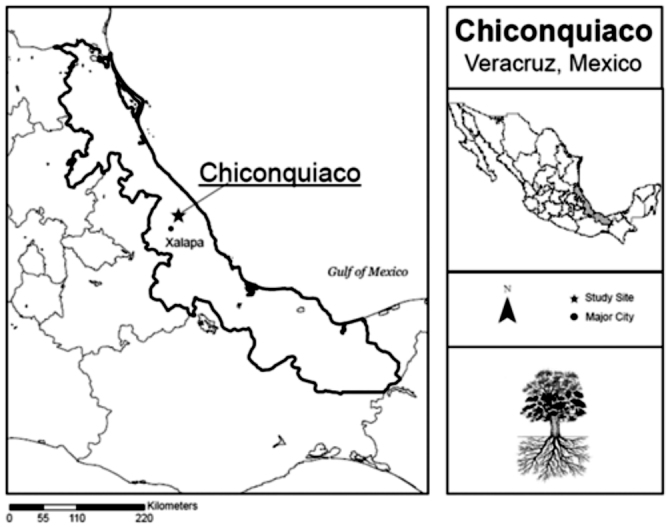
The study was conducted in tropical montane forest near the town of Chiconquiaco, in the state of Veracruz, Mexico. High quality figures are available online.

The study was conducted in tropical montane forest near the town of Chiconquiaco, in the state of Veracruz, Mexico (Figure 4). The area has three seasons: moderately dry and cool from October to March, dry and warm from April to May, and wet and warm from June to September. Mean annual temperature is 15.2° C and total mean annual precipitation is 1532 mm) (Williams-Linera et al. 2000). The area was covered by heavy fog on most days, and humidity levels typically remain high even during the relatively dry months. The elevation was approximately 2000 m, and the habitat was a mosaic of mature forest patches, cattle pastures, and small cornfields. The study trees were located in two ∼ 25 ha forest patches and two ∼ 35 ha pasture areas all on west-facing slopes. All sites were separated by at least 1 km. Forest areas had closed canopies dominated by *Quercus* spp., and pasture areas were open and included a few, large, scattered *Quercus* spp. individuals. Forest fragments had low edge to interior area ratios and were comprised of a canopy of hybridizing assemblages of *Q. laurina* Bonpl., *Q. germana* Schltdl. & Cham., *Q. salicifolia* Née, *Q. corrugata* Hook., *Q. affinis* Schweid., and *Q. xalapensis* Bonpl., all of which are capable of hosting the scale insect *S. garmilleri.*

### Scale insect density measures

For determination of scale-insect densities, insects were counted on oak trees in nine randomly chosen 10 × 10 m plots; three plots each in forest interior, forest edge, and pasture. Though it is difficult to determine species identity accurately with vegetative characters and complications of hybridization, all oak trees on which scale insect density was recorded were presumed to be *Quercus laurina.* In these plots, the diameters at breast height of all *Q. laurina* trees over 2 m tall were recorded. Anal tube density was determined as number of filaments occurring within copper wire frames; 20 × 20 cm for trunks and large branches, 10 cm × 40 cm for thinner branches. Insect counts within these wire frames were conducted at 1m, 2m and 3m on the north, south, east, and west sides of the tree for a total of 12 measurements per tree. Other studies in New Zealand have shown that 90% of filaments are connected to feeding larvae (Moller and Tilley 1989), so anal-tube presence was taken as a proxy for insect presence. It is not specifically known whether the presence of an anal filament indicates that *S. garmilleri* is actually alive. Anal filaments producing honeydew were assumed to be attached to living insects. Filaments without drops may still be connected to a living insect, albeit not producing honeydew at the time. The number of filaments with honeydew drops within each frame was also recorded. To determine the overall insect density, we used the sum of the 12 insect counts per each 400 cm^2^ sample frame for a total of 4800 cm^2^ sampled. On the basis of diameters at breast height measurements, these trees were categorized as small (5–28.6 cm), medium (29–46.3 cm), or large (48.9–83 cm); each category included roughly equal numbers of trees when averaged over the three habitats. Pasture areas only included small and medium sized trees, whereas forest and forest edge habitats included oaks in all size classes. Insect density data were not normally distributed, but population distributions were uniform among samples. Kruskal—Wallis nonparametric comparison tests were used to determine whether tree height, aspect, and tree-diameter size class affected density of scale insects. Multiple comparison tests were conducted on pair wise differences among groups. Type I error across tests was controlled by the Holm's sequential Bonferroni approach.

### Anal filament and honeydew drop measures at observation trees

Forty observational trees were chosen for the study; ten at each of two forest sites and two pasture sites. All sites were separated by at least 1 km. All observation trees harbored colonies of scale insects producing honeydew, although not every tree at each site had such colonies. Each individual tree was observed on four occasions, divided evenly among morning and afternoon periods between March 2002 and May 2002. Observation times for each tree were randomized. At each tree, 10 scale insects producing honeydew on the lower trunk of the observation tree were randomly chosen, and sugar concentration and volume of the exposed honeydew drop were recorded in addition to the anal filament length (n = 1600). Honeydew drop volume was measured with 15 µl microcapillary tubes (Drummond Scientific Company, www.drummondsci.com). A hand-held refractometer (Bellingham & Stanley, www.bellinghamandstanley.com) designed for volumes as small as 0.5 µl was used to measure sugar concentration in honeydew. The presence of amino acids in honeydew may contribute to the refractive index, but this effect was considered negligible (Inouye et al. 1980). Refractivity is a reliable measure of the sugar concentration of honeydew drops (Grant and Beggs 1989). Only honeydew from ground level to 3 m was sampled. Ambient air temperature and relative humidity were also recorded when measurements were made. Lengths of scale insect anal filaments were measured using a digital caliper.

Independent-samples *t*-tests were used to test for differences in anal filament length by habitat type. Correlation analysis was used to examine the relationships among sugar concentration, honeydew volume, relative humidity, temperature, and anal-tube length.

### Anal filament growth rate at observation trees

Half of the original 40 observation trees were randomly chosen as experimental trees for examination of anal tube growth rate. At each tree 12 scale insects were individually marked with colored pins just above their position on the tree. The anal filaments originating from scale insects were measured using digital calipers and were then experimentally removed on 25 April 2002. Only insects producing honeydew drops at initiation of the experiment were chosen for study to ensure that living insects were marked. The following day anal filaments were re-measured and average growth over a 24 hour time period was calculated. Correlations between the original length of each anal tube and its rate of re-growth were calculated. Independent-samples *t*-tests were used to test for differences in tube growth between pasture and forest. Some originally marked insects were not recovered during the re-measurement period due to pin drop from oak trunks.

## Results

### Scale insect density measures

The overall mean number of anal filaments per 400 cm^2^ frame was 13.4 (± 20.5 SD). The mean percentage of productive scale insects (filaments with honeydew drops/total number of anal filaments) at the time of sampling per 400 cm^2^ was 36.4% (± 34.2 SD). Because the presence of a honeydew drop may be influenced by wind or rain, this figure may be an underestimate. We therefore used anal-tube densities as the measure of scale insect abundance.

**Figure 5.**
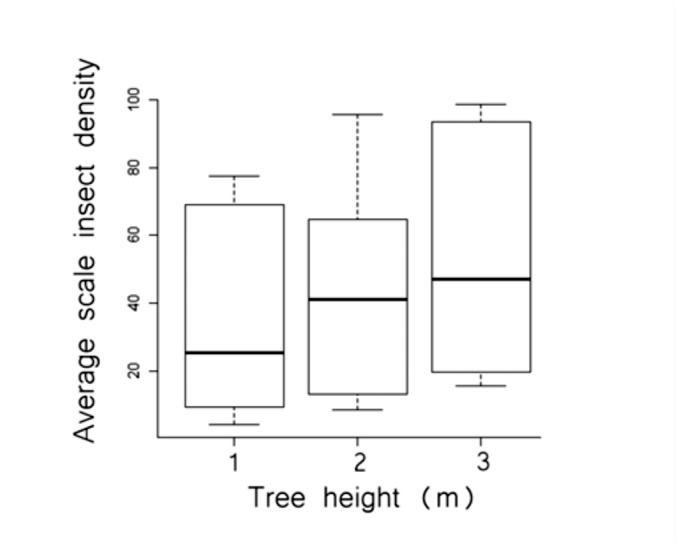
Mean estimates (95% confidence interval, standard deviation) of scale-insect density per 400 cm^2^ on trees at different heights in forest habitat. High quality figures are available online.

We found no significant difference in anal tube density among the four aspects (north, south, east, and west) in any habitat (*p* > 0.05 in all cases; Kruskal-Wallis test). The density of insects among the three trunk heights was not significantly different in edge habitat (χ^2^ = 0.70, *p* = 0.71) or in the pasture area (χ^2^ = 0.001, *p* = 0.99), although there was a significant difference in forest habitat (χ^2^ = 10.98, *p* < 0.01). Trees in the forest had greater densities of scale insects at three meters height in comparison to lower heights (Figure 5).

**Figure 6.**
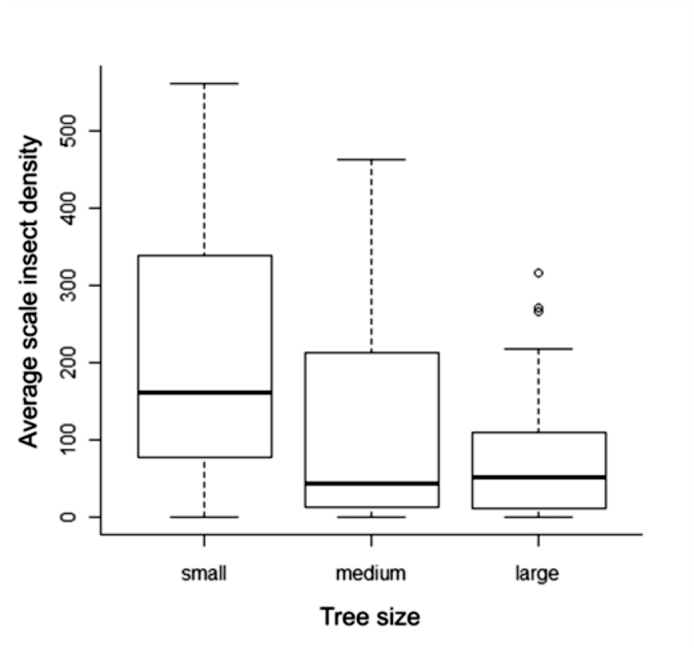
Mean estimates (95% confidence interval, standard deviation) of scale-insect density per 400 cm^2^ on trees of different size classes within forested habitat. Small = 5–28.6 cm, medium = 29– 46.3 cm, large = 48.9–83 cm diameters at breast height. High quality figures are available online.

Anal-tube density was highest in the pasture area (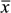 = 16.5, ± 30.9 SD), followed by edge trees (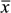 = 14.8, ± 18.4 SD) and forest trees (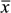 = 10.5, ± 14.9 SD). These differences were not significant (*p* > 0.05 in all cases; Kruskal-Wallis test). The three tree diameter classes differed significantly in median scale-insect densities (χ^2^ = 7.91, *p* < 0.05). Smaller trees harbored greater densities of scale insects than did medium and large trees (Figure 6). Multiple comparison tests revealed the small and medium diameters at breast height classes differed significantly (*p* < 0.05), as did the small and large classes (*p* < 0.05). The medium size class did not differ significantly from the large class (*p* = 0.831).

### Anal filament and honeydew drop measurements

On average, scale insects associated with forest trees had longer anal filaments than those on forest edges or in pastures. Independent-samples *t*-tests revealed that habitats differed significantly in scale insect anal-tube length; mean tube length was on average 5.2 mm greater in forest than in pasture areas (Table 1). Habitats did not differ significantly in sugar concentrations or in the volume of honeydew (Table 1).

**Table 1.**
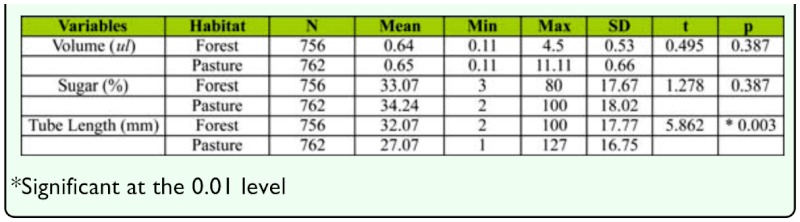
Means, standard deviations, minimum and maximum values for honeydew-drop volume, sugar concentrations, and anal-tube lengths for scale insects (*Stigmacoccus garmilleri*) on oaks in forest (n = 756) and pasture habitats (n = 762) of Chiconquiaco, Mexico. Independent-samples t-tests revealed that habitats differed significantly in anal-tube length; however habitats did not differ significantly in sugar concentrations or volume of honeydew.

**Table 2.**
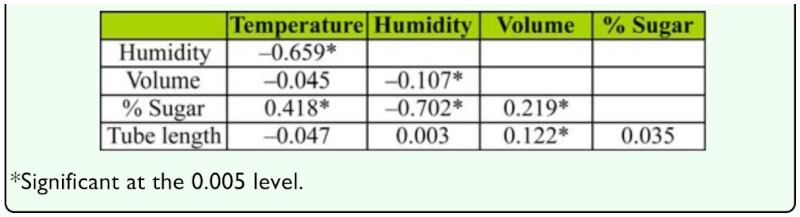
Spearman's correlation coefficients were calculated for the predictive strength of the recorded variables (temperature, relative humidity, drop volume, sugar concentration, and anal-tube length). Data were combined from 20 forest trees and 20 trees located in pasture habitat in Chiconquiaco, Mexico. In all, 1518 insects producing honeydew were examined, and data on temperature and humidity were recorded when sugar concentration, drop volume, and tube length were measured.

**Figure 7.**
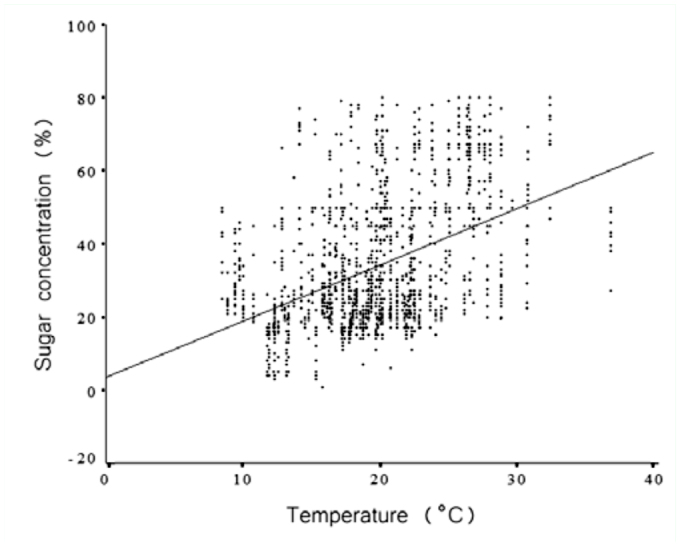
Honeydew sugar concentration plotted against temperature recorded at time of measurement. Data were collected from 20 trees in forest habitat and 20 trees located in pasture habitat in Chiconquiaco, Mexico, n = 1518 insects producing honeydew were recorded between 15 March 2002 and 17 April 2002. High quality figures are available online.

**Figure 8.**
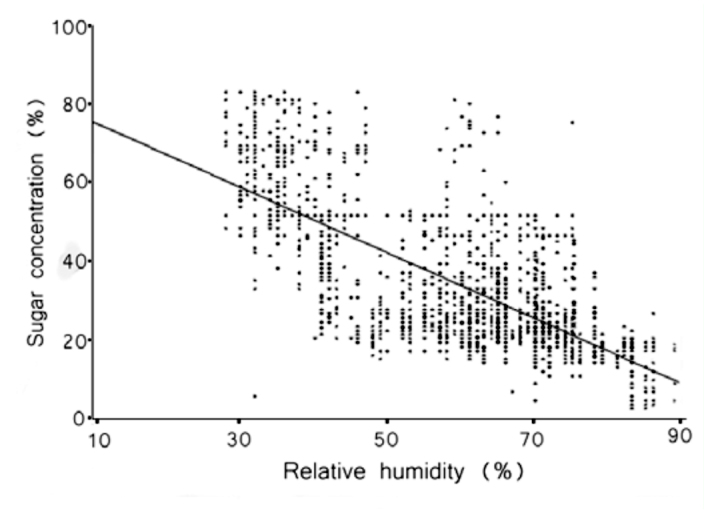
Honeydew sugar concentration plotted against relative humidity recorded at time of measurement. Data were collected from 20 trees in forest habitat and 20 trees located in pasture habitat in Chiconquiaco, Mexico, n = 1518 insects producing honeydew were recorded between 15 March 2002 and 17 April 2002. High quality figures are available online.

Correlation analysis was used to examine the relationships among sugar concentration, honeydew volume, relative humidity, temperature, and anal-tube length (Table 2; Figures 7, 8). The Bonferroni approach was used to control for Type I error across the 10 correlations; a *p* value of less than 0.005 was required for significance. The results show that 6 out of 10 correlations were statistically significant. Honeydew-drop sugar concentrations were positively correlated with increases in temperature (Figure 7) and negatively correlated with relative humidity (Figure 8).

### Anal filament growth

Prior to removal of anal-tube filaments, scale insects used for measurements of anal-tube regrowth had a mean anal-tube length of 33.56 mm, with a large standard deviation of 20.84 mm. Twenty-four hours after anal filaments were removed the new filaments had reached a mean length of 3.89 mm (± 1.59 SD). An independent-sample *t*-test, not assuming equal variances, revealed that that forest and pasture trees differed in both original mean tube length and mean tube length 24 hours after tube removal. Insects in forest trees had significantly longer original anal filaments than did those in pasture trees (t_115_ = -3.81, *p* < 0.01), and those in pasture trees had significantly higher rates of anal-tube regrowth (t_193_ = -2.09, *p* < 0.05). The correlation between rate of regrowth and original length was low (Pearson correlation coefficient; *r* = 0.056) and not statistically significant (*p* = 0.416).

## Discussion

Data reported here reveal differences in the biology of scale insects depending on habitat type. In Chiconquiaco and in many other areas of Mexico, highland tropical forest habitat exists in a landscape dominated by pasture. The maintenance of key food resources like scale-insect honeydew in these disturbed habitats can be important to maintaining the animal community and critical landscape-level processes.

### Scale insect distribution

Knowledge of the spatial distribution and abundance of *S.* *garmilleri* within host trees is essential to understanding how land-use change can affect biotic insect-plant interactions, which in turn can modify forest dynamics. Populations of *S.* *garmilleri* are ideal for testing within-tree heterogeneity in herbivore densities because feeding instars are embedded and sedentary within the bark of the tree, and their spatial distribution is not a transitory phenomenon as it would be in the evaluation of free-living herbivores. There is strong evidence of a stationary spatial distribution in beech scale insects, which share similar natural history characters with *S. garmilleri*, over time periods of several months to one year (Kelly et al. 1992; Murphy and Kelly 2003).

Variables that might affect the distribution of scale insects within single trees include altitude, host species, exposure to sunlight, aspect (north, south, east, west), trunk diameter, and possibly region (Kelly 1990; Dungan and Kelly 2003). Crozier (1981) found higher densities of scale insects on northern aspects of *Nothofagus solandri* trunks and hypothesized that higher mean temperatures on this side contributed to the effect. In agreement with Kelly (1990), aspect was not correlated with anal-tube density in our study. The varying slopes and corresponding microclimate created on tree trunks, irrespective of aspect, may have confounded the findings in this study.

Scale insect densities have been reported to be higher on edge-habitat trees in *Nothofagus* forests of New Zealand, although these differences were not quantified (Crozier 1981). In the present study, edge trees had greater densities of scale insects than forest areas, and densities were highest in pasture areas. Wardhaugh et al. (2006) recognized the greatest densities occurring occurred on bark surfaces in the canopy rather than on the trunk and on the lower rather than upper sides of the branches. Our findings suggest that trees in pasture areas and on the forest edge have dense colonies at all heights measured, whereas scale insect colonies in the forest interior had significantly greater numbers of scale insects at the highest height measured.

Tree size and scale insect densities were inversely related with small trees harboring the highest densities, supporting Wardhaugh (2006) who noted a decrease in scale insect density with increasing diameters at breast height or branch diameter. In contrast, Kelly (1990) found that intermediate-sized trees harbored significantly greater tube densities while the small trees and thin upper branches bore almost no scale insects. Even the thinnest branches of oak trees in Chiconquiaco were found to frequently harbor scale insects. Physiological differences between the insect species and/or tree species in the distinct geographic areas of New Zealand and Mexico may cause this difference.

Wardhaugh et al. (2006) discuss how insect density differences in relation to tree size may be driven by a positive correlation between bark thickness and diameter. A number of studies have found that the establishment of sap-sucking insects was influenced by bark characteristics (Geraud-Pouey et al. 2001). Oak trees that supported no scale insects on the trunk likely had a low availability of establishment sites. It is possible that newly created fissures were inhabited by scale insects because they were swiftly colonized by crawlers, the dispersal stage of *S.* *garmilleri.* Processes that result in cracking of the bark surface such as increased exposure to the sun (Nicolai 1986) or rapid/released growth are both processes that are more predominant after deforestation of nearby trees. Forest loss and land use change may increase the number of available establishment sites within remaining trees.

### Honeydew volume/concentration

The highest honeydew sugar concentrations were found when humidity was low and temperature high. In warm sun, droplets become more concentrated as water evaporates. In forests of New Zealand, morning sugar concentrations were lower because less water had evaporated from the honeydew. Honeydew was more concentrated on hot days with dry winds (Dungan and Kelly 2003). This was not always the case in our study area; afternoons were often characterized by an increase in fog and mist and a decrease in temperature. A dynamic model by James et al. (2007) of honeydew droplet production by sooty-beech scale insects predicts different types of behavior depending on local environmental conditions. Honeydew droplets are highly concentrated in dry conditions with high evaporation rates, complicating further excretion resulting in cessation of droplet formation. In humid conditions, droplet formation continues indefinitely (James et al. 2007).

Dungan et al. (2007) found that rates of production were significantly related to environmental conditions over the hours preceding measurement, with air temperature and air saturation deficit averaged over the preceding 24 and 12 hours, respectively. The contribution of temperature and humidity to variability in honeydew production illustrates a strong influence of environmental conditions. On average, pasture areas were warmer and less humid than forested areas. The lack of significant differences in this study among habitats in honeydew sugar concentration and volume does not rule out effects by changes in land management practices on honeydew production by scale insects. Due to the temporal variability of these environmental conditions, monitoring insects and honeydew production simultaneously in the varying habitats is suggested for future studies.

### Anal filaments

The finding of significantly longer anal filaments in forest trees compared to pasture trees may have been due to greater exposure of the pasture trees to winds and rain, which can break filaments. Filaments also may be broken by wasps (Moller and Tilley 1989) or birds (Greenberg et al. 1993; Latta et al. 2001) feeding on the honeydew. Filament breakage inside protective netting placed around *S.* *garmilleri* colonies in Chiapas, Mexico, was only 30%,compared to 70% in an unprotected sample during the same period (Greenberg et al. 1993). In Chiconquiaco birds occasionally broke filaments while feeding on honeydew, and cows were observed rubbing against pasture trees, contributing to breakage low on the tree trunks in pasture areas.

In the present study, re-growth of broken filaments on scale insects occurred at an average rate of 4 mm per 24 hours, a previously undocumented phenomenon. A tube of average length can therefore be replaced within about seven days, assuming a constant growth rate. Breakage is apparently not detrimental to the insects; they can produce a tube long enough to secrete honeydew drops within 24 hours. The higher rate of tube growth in pasture trees than in forest trees may be attributable to higher temperatures and greater sunlight availability.

## Conclusions

Scale insect honeydew is an important food source for many organisms inhabiting montane cloud forests (Gamper and Koptur 2010). Honeydew is particularly essential for nectar feeding organisms when nectar resources are scarce (Murphy and Kelly 2003). Changes in availability and distribution of honeydew could have profound effects on a large community of other organisms.

Examination of the distribution of scale insects in this system indicates that mosaics of forest and pasture provide good habitat for *S.* *garmilleri.* The great abundance of honeydew on scattered pasture trees may be of particular importance in this region. Scattered pasture trees have been found to be keystone structures because of the disproportionally large ecosystem services they provide relative to the area they occupy, in addition to the maintenance of habitat and connectivity to other habitat types (Manning et al. 2009). Scattered trees are threatened in many places, making appropriate levels of tree regeneration and preclusion of premature mortality of mature trees essential for maintaining these trees within landscapes (Gibbons et al. 2008). Despite the benefits of habitat and food resources to other organisms, increases of scale insects on scattered pasture and forest edge trees may induce physiological stress, transform forest growth dynamics, and decrease reforestation potential (Gamper, in prep).

The spatial distribution of scale insect populations on trunks and branches of trees of increasing diameters at breast height may indicate a strong temporal component to the spatial dynamics of scale insects driven by changing host tree phenology. Future studies on phytophagous insects infesting large host trees should consider more explicitly changes in population dynamics both spatially and temporally (Wardhaugh et al. 2006). In order to detect interactions between tree size and location (interior, edge, pasture), future experimental design should incorporate information on geographic location, with accompanying scale insect density and other descriptive attributes for a thorough, spatially-explicit statistical analysis.

Understanding more about the dispersal of scale insects and potential genetic predisposition for some individual trees to be more susceptible to scale insect populations may also be fundamental in understanding how they will be distributed in a changing landscape, and how the modifications of the landscape will alter their distribution. In cases of dispersal limitation, isolated pasture trees could lead to deleterious changes in the insect population genetic structure. These potential directions for future research may yield insight into unanswered questions about this important food resource in tropical montane cloud forests.
